# Identifying causal associations between women’s reproductive traits and risk of schizophrenia: a multivariate validated two-sample Mendelian randomization analysis

**DOI:** 10.1186/s12888-024-05614-5

**Published:** 2024-02-24

**Authors:** Wenxi Sun, Xiaohui Wu, Haidong Yang, Shiting Yuan, Jun Chen, Yiru Fang, Xiaobin Zhang

**Affiliations:** 1 Suzhou Guangji Hospital, Suzhou, Jiangsu Province; Affiliated Guangji Hospital of Soochow University, Suzhou, 215137 Jiangsu Province China; 2 Clinical Research Center & Division of Mood Disorders, Shanghai Mental Health Center, Shanghai Jiao Tong University School of Medicine, Shanghai, 200030 China; 3 Department of Psychiatry, The Fourth People’s Hospital of Lianyungang, The Affiliated KangDa College of Nanjing Medical University, Lianyungang, 222003 People’s Republic of China; 4 Department of Psychiatry & Affective Disorders Center, Ruijin Hospital, Shanghai Jiao Tong University School of Medicine, Shanghai, 200025 China

**Keywords:** Women’s reproductive traits, Schizophrenia, Mendelian randomization, Causal relationship

## Abstract

**Background:**

A significant association between women’s reproductive traits and the risk of schizophrenia (SCZ) has been discovered, but the causalities remain unclear. We designed a two-sample univariate Mendelian randomization (MR) study using female-specific SNPs collected from a large-scale genome-wide association study as a genetic tool to explore the causal effect of female reproductive traits on the risk of SCZ, and conducted a multivariate MR study to re-validate the above findings.

**Methods:**

From extensive genome-wide association studies (GWAS) of people with European ancestry (*n* = 176,881 to 418,758 individuals), summary-level data on five female reproductive variables were extracted. Summary-level information on SCZ was taken from a GWAS meta-analysis involving 320,404 people with European ancestry. The inverse variance weighting estimations for both univariable MR (UVMR) and multivariable MR (MVMR) were presented as the primary results. MR-Egger, weighted median, simple mode, and weighted mode regression methods for UVMR, and MVMR-Egger, MVMR-Lasso, and MVMR-median methods for MVMR were used for sensitivity analyses.

**Results:**

The UVMR produced compelling proof for a connection between genetically predicted later age at first sexual intercourse (AFS) (OR, 0.632; 95% CI, 0.512–0.777; *P* < 0.01) and decreased SCZ risk. Pleiotropy analysis of the AFS-SCZ association confirmed the robustness of the MR results (*P* > 0.05). Consistent, substantial causal effects of AFS (OR, 0.592; 95%CI, 0.407–0.862; *P* < 0.01) on the risk of SCZ were demonstrated after adjusting for body mass index, years of schooling, and smoking initiation using MVMR.

**Conclusions:**

Our findings provide convincing evidence that early AFS is a risk factor for SCZ. SCZ risk may be decreased by raising awareness of reproductive healthcare for women.

**Supplementary Information:**

The online version contains supplementary material available at 10.1186/s12888-024-05614-5.

## Background

Over the past three decades, the number of people struggling with mental illness has risen rapidly, from 80.8 million to 125.3 million [1]. Mental illnesses, in particular, place a sizeable economic burden on families and society [2], and schizophrenia (SCZ) is considered the most serious of all. SCZ is a heterogeneous disorder that includes positive symptoms (e.g., delusions and hallucinations), negative symptoms (e.g., flat mood and lack of motivation), and cognitive symptoms (e.g., decreased executive functioning) [3]. Many people with this disorder do not fully recover, and those who enter periods of remission often experience shame and social isolation [4, 5]. Compared to male patients with SCZ, female patients have higher rates of insomnia and poorer performance on several dimensions of cognition including visuospatial/structural and language [5]. Aside from conventional risk factors for SCZ, emerging evidence has identified additional sex-specific risk factors for women related to female reproductive traits [6, 7].

Women’s reproductive traits, such as age at first birth (AFB), age at first sexual encounter (AFS), age at menarche (AAM), age at last live birth (ALB), and age at menopause (AMP), have a significant bearing on a population’s capacity for evolutionary adaptation and later-life health [8]. Numerous observational studies [9–12] have found that children with younger or older parents are more likely to have a variety of mental health problems than children of average-aged parents, with a particular emphasis on the risk of SCZ in children related to parental age. In addition, people with mental illness and their relatives may be more likely to engage in risky and impulsive behaviors that can lead to premature sexual intercourse or early pregnancy and childbirth in women [13]. Moreover, age at menopause and at menarche are frequently closely linked to mental health risks [14, 15]. In addition to epidemiological findings, the phenotypic association between female reproductive features and the risk of SCZ may have a genetic foundation [16–19]. Nonetheless, it is unclear whether these variations in female reproductive traits increase the likelihood of developing SCZ or whether they are all caused by other underlying evidence.

Concerning the rapid growth of genome-wide association studies (GWAS), Mendelian randomization (MR) analysis applying single nucleotide polymorphisms (SNPs) closely connected to phenotypes as instrumental variables (IVs), is becoming increasingly commonplace [20]. Considering SNPs are given at random during pregnancy and always predate illness manifestation, MR findings are less subject to confounding and reverse causation, which are key drawbacks of traditional observational research [21]. As a result, there is reason to suppose that the results of MR are stable and convincing to some extent. Guiyan Ni et.al [22] recently discussed the relationship between six psychiatric disorders and female reproductive traits using a two-sample MR method, and they found an evident causal association between attention-deficit/hyperactivity disorder and female reproductive traits (AFB, AFS, & AMP). With the massive update and public availability of the GWAS database on various clinical and subclinical aspects of psychiatric disorders, a new round of discussion on women’s health and psychiatric disorders has been initiated [23–25]. A causal association between female reproductive traits (AFB, AFS, &AAM) and major depression has recently been shown through a scientific design using MR methods [26]. Notably, it also provides new opportunities to explore the relationship between female reproductive traits and the risk of SCZ.

In this study, using just-available summary genetic association statistics from large-scale GWASs, we performed a two-sample MR study to explore the causal associations between women’s reproductive traits (AFB, AFS, AMP, ALB, & AAM) and the risk of SCZ. We utilized multivariable MR for evidence that women’s reproductive traits had a substantial causal influence on the risk of SCZ, independent of indicated modifiable risk factors. Our aim was to gain a comprehensive understanding of the impact of reproductive factors on SCZ and to re-evaluate the results from a genetic perspective.

## Methods

### Study design

**Fig. 1:**
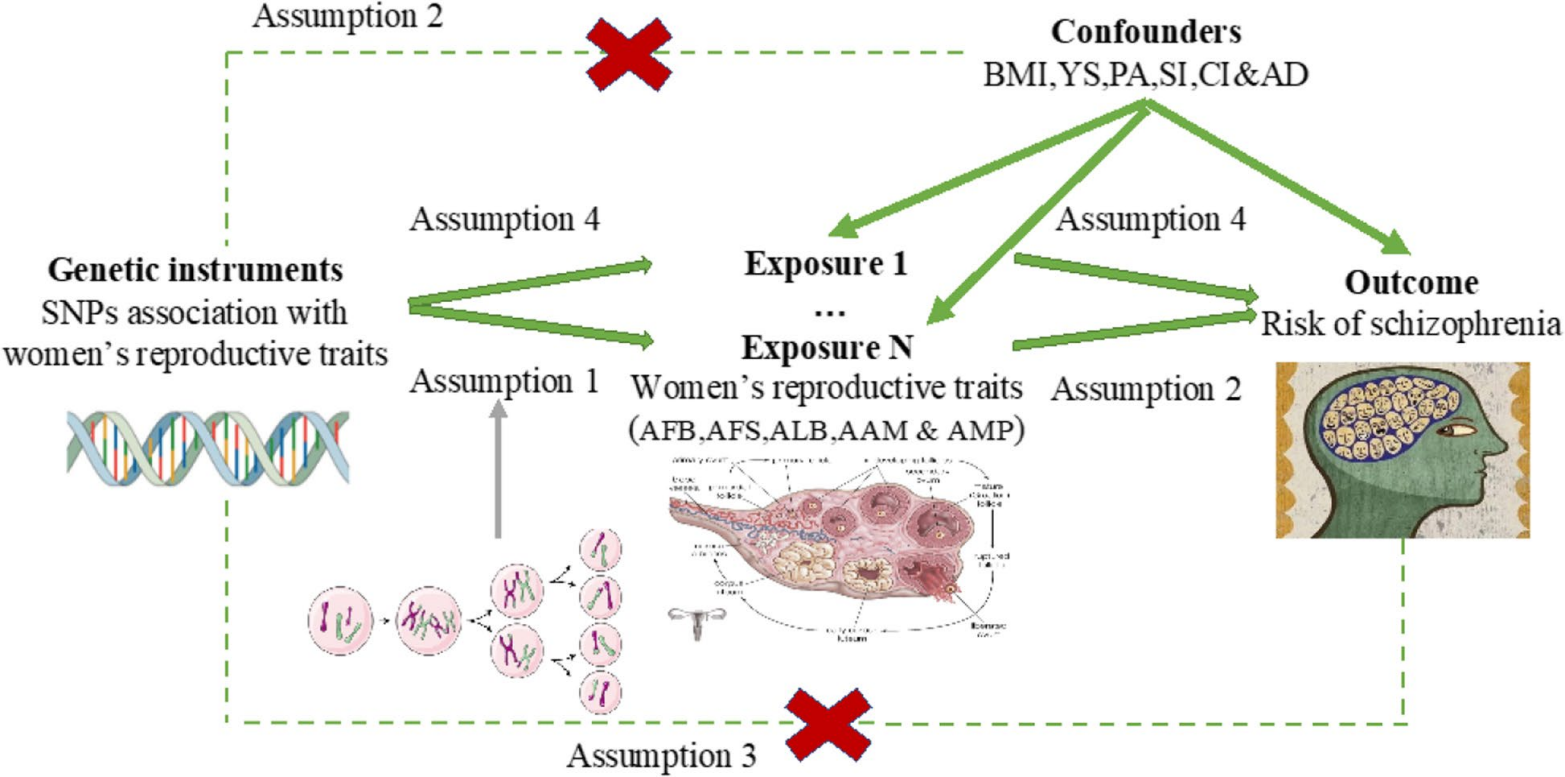
Diagram for Mendelian randomization (MR). MR was developed on the premise of three assumptions. First, SNPs designated as instrumental variables (IVs) should be significantly associated with the exposure (Assumption 1). Second, SNPs selected as IVs are required to be independent of confounders (Assumption 2). Third, rather than being directly correlated, the relationship between IVs and the risk of SCZ (outcome) only occurs vis-a-vis reproductive variables (exposure) (Assumption 3). Fourth, the first assumption in the MVMR (Assumption 4) is that genetic variations are caused by one or more of the exposures. BMI, body mass index; YS, years of schooling; PA, physical activity; SI, smoking initiation; AD, alcoholic drinks per week; CI, coffee intake

A brief description of the two-sample Mendelian randomization (MR) designs is displayed in Fig. 1. To thoroughly investigate the connections between five women’s reproductive traits on the risk of SCZ, we conducted two-sample univariable MR (UVMR) and multivariable MR (MVMR). UVMR rests on three main assumptions: (1) The exposure has a substantial correlation with the genetic variation determined as the instrumental variable (Assumption 1); (2) The genetic variation in discussion is not linked to any confounding factors (Assumption 2); (3) Genetic variation influences outcome only through exposure, not via other pathways (Assumption 3) [27]. In comparison to the UVMR assumptions, the initial assumption of MVMR (Assumption 4) was the genetic variations related to one or more of the exposures, whereas the remaining assumptions were consistent with UVMR [28]. First, we chose genetic variations for each woman’s reproductive trait. Second, we used MVMR models to evaluate the direct influence of reproductive variables on the risk of SCZ while adjusting for the modifiable risk factors.

### Data sources

The exposures in the present analysis included women’s reproductive features such as AFB, AFS, AAM, ALB, and AMP. The research outcome was the risk of SCZ. Summary-level data (effect estimates, standard errors, and *P*-values) for each trait were acquired from recent major European GWASs (Table 1).

**Table 1:** Data sources used in the MR analyses for the current study

Phenotype	Participants included in analysis	Ancestry	Unit	Cohorts/consortium	PMID and/or web link
**Exposures**					
AFB	418,758 females	European	Year increase in AFB	36 studies	34,211,149 https://www.ebi.ac.uk/ gwas/studies/GCST90000050
AFS	214,547 females	European	SD increase in AFS	36 studies	34,211,149 https://www.ebi.ac.uk/ gwas/studies/GCST90000047
AAM	182,416 females	European	Year increase in AAM	ReproGen	25,231,870 https://www.reprogen.org/data_download.html
ALB	184,996 females	European	SD increase in ALB	MRC-IEU UK Biobank	https://gwas.mrcieu.ac.uk/datasets/ukb-b-8727/ http://www.nealelab.is/uk-biobank
AMP	176,881 females	European	SD increase in AMP	MRC-IEU UK Biobank	https://gwas.mrcieu.ac.uk/datasets/ukb-b-17422/ http://www.nealelab.is/uk-biobank
**Outcome**					
Risk of SCZ	320,404 individuals (76,755 cases and 243,649 controls)	Mixed(74.3% were European)	logOR	PGC consortium	35,396,580 10.6084/m9.figshare.19426775
**Confounders**					
BMI	461,460 individuals	European	SD	MRC-IEU UK Biobank	https://gwas.mrcieu.ac.uk/datasets/ukb-b-19953/ http://www.nealelab.is/uk-biobank
YS	766,345 individuals	European	Years	SSGAC consortium	30,038,396 https://thessgac.com/papers/
PA	460,376 individuals	European	SD	MRC-IEU UK Biobank	https://gwas.mrcieu.ac.uk/datasets/ukb-b-8764/ http://www.nealelab.is/uk-biobank
SI	607,291 individuals	European	standardized log odds	GSCAN consortium	30,643,251 https://genome.psych.umn.edu/ index.php/GSCAN
AD	335,394 individuals	European	SD	GWAS and Sequencing Consortium of Alcohol and Nicotine use	30,643,251
CI	428,860 individuals	European	SD	UK Biobank	http://www.nealelab.is/uk-biobank http://gwas.mrcieu.ac.uk/datasets/ukb-b-5237

*AFB* Age at first birth, *AFS* Age at first sexual intercourse, *ALB* Age at last live birth, *AAM* Age at menarche, *AMP* Age at menopause, *BMI* Body mass index, *YS* Years of schooling, *PA* Physical activity, *SI* Smoking initiation, *AD* Alcoholic drinks per week, *CI* Coffee intake, *SCZ* Schizophrenia

### Women’s reproductive traits

Mills et al. [29] carried out the largest GWAS meta-analysis which incorporated the data of 418,758 females for AFB, obtained from a total of 36 studies of European origin. AFB was measured as a continuous measurement for all women who had ever given birth. AFS genetic variations were also discovered in the largest GWAS, which included 214,547 females of European ancestry delivery from the UK Biobank [29].

AAM genetic variations were discovered by a GWAS meta-analysis which incorporated the data of 182,416 females of European ancestry, obtained from 57 studies in the ReproGen collaboration [30]. Each study incorporated autosomal SNP values that passed quality control procedures (including minor allele frequency > 1%) by meta-analysis and satisfied genome-wide significance levels in connection with age at menarche (*P* < 5 × 10^−8^).

The UK Biobank work presented summary-level data on age at last live birth and age at menopause (http://www.nealelab.is/uk-biobank). In the UK Biobank, the age at last live birth and at menopause are the age at which a woman gave birth to her last child and at which her menstruation ceased, respectively. The database includes the age at last live birth for 184,996 women and the age at menopause for 176,881 women. During the touch-screen phase, participants self-reported their age at last live birth and age at menopause. In addition, we utilized the second round of Neale Lab’s GWAS (https://gwas.mrcieu.ac.uk/datasets/). Genetic associations were adjusted for 20 genetic principal components as well as age [31].

### Risk of schizophrenia

We utilized the Psychiatric Genomics Consortium’s (PGC) (https://pgc.unc.edu) GWAS for SCZ, which, at the study period, was the largest publicly accessible GWAS, involving 320,404 individuals of European ancestry (76,755 cases and 243,649 controls) [32]. Based on the UK Biobank data, the regression model was adjusted for gender, age, genotyping array, and the first eight major components of population structure. The inverse variance weighted method was applied to meta-analyze the summary statistics from the dataset to determine the relationships between SNPs and SCZ.

### Selection of genetic instrumental variables

All SNPs for UVMR and MVMR are required to be firmly and independently predicted exposures from the published GWAS at genome-wide significance (*P* < 5 × 10^−8^) in order to comply with the MR assumptions (Fig. 1). We evaluated whether any of these SNPs were linked with covariates (body mass index, BMI; years of schooling, YS; physical activity, PA; smoking initiation, SI; alcoholic drinks per week, AD; coffee intake, CI) and outcome (risk of SCZ) at a *P*-value of 5 × 10^−8^ for UVMR by applying publically available GWAS summary data. In the UK Biobank, we utilized the second round of Neale Lab’s GWAS (https://gwas.mrcieu.ac.uk/datasets/). We collected SNPs corresponding to BMI, PA, and CI by meta-analyzing GWAS from approximately 461,460, 460,376, and 428,860 adult populations of European ancestry, respectively (http://www.nealelab.is/uk-biobank). A meta-analysis of GWASs on roughly 1.1 million European ancestors supplied by the prior meta-analysis of the Science Genetic Association Consortium (SSGAC) yielded genetic connections with YS [33]. In publications with sample sizes of up to 1.2 million participants, genetic correlations with other possible confounders (SI and AD) were also found. Table 1 provides extensive information on these investigations [34]. As with UVMR, we evaluated whether any of these SNPs were linked to confounders (BMI, YS, PA, SI, AD, and CI) and outcome (the risk of SCZ) in the MVMR. When the number of suitable instrumental variables is limited to 10 or fewer, the IV selection threshold should be relaxed to a *p*-value threshold of 1 × 10^−6^.

In addition, the F-statistic serves as a measure of the strength of instruments in predicting one exposure. It is applied to individual or summary-level data as well as to univariate or multivariate MR estimates. The mean F-statistic is calculated by summing two or more F-statistics associated with the exposure and subsequently computing their average. Hence, we calculated the mean F statistics of SNPs to evaluate their importance for UVMR [35]. The mean F-statistic was greater than 10, indicating that the SNPs for the UVMR exposures were valid. The conditional F statistic is a measurement instrument for predicting the strength of the effect of one exposure with the conditioned exposure on other exposures. The conditional F-statistic to measure the strength of SNPs for MVMR was not calculated when two samples were overlapped since the required pairwise covariances between SNP associations are only determinable using individual-level data [36].

### Statistical analysis

SNPs that had been excluded in the outcome datasets were substituted with proxies in LDlink (https://ldlink.nci. nih.gov/) where linkage disequilibrium (LD) R^2^ was greater than 0.001 within 10 Mb. To identify the causal relationship between women’s reproductive characteristics and the risk of SCZ, we carried out UVMR on the data after extraction and harmonization. In the primary analysis, we determined a Wald ratio estimate for each genetic variation and used the inverse-variance weighted (IVW) approach to compile the estimates. A straightforward estimate is provided by the IVW with the multiplicative random effects method, which additionally takes into account any potential heterogeneity in the Wald ratio estimations from SNPs [37]. Therefore, random-effects IVW models are applied when there is heterogeneity; otherwise, the fixed-effect IVW model is used. We also conducted sensitivity analyses employing techniques with various assumptions for horizontal pleiotropy, such as MR-Egger regression, weighted median, simple mode, and weighted mode regression method, in order to evaluate the robustness of our findings. The MR-Egger intercept was examined to determine whether horizontal pleiotropy existed [38]. The Cochran Q test and I^2^ index were used to analyze the heterogeneity of causal impact estimates across every variation for each reproductive characteristic in women.

Previous studies have confirmed that BMI [39], CI [40], AD [40], SI [41], PA [42] and YS [43] are strongly associated with SCZ. Adjusted for variable risk factors, the MVMR analyses were performed to explore the influence of women’s reproductive factors on genetic susceptibility to developing SCZ. By executing MVMR-IVW and choosing random effects or fixed effects depending on heterogeneity as mentioned in UVMR, we extended the IVW MR method for MVMR. The MVMR-Egger, MVMR-Lasso, and MVMR-Median were employed for the sensitivity analyses. To account for both measurable and unmeasured pleiotropy, the MVMR-Egger intercept method was devised.

The results for the effect of AFS, ALB, AMP, AAM, and AFB on SCZ risk are presented as OR (95% CI). The Bonferroni method was used to correct for multiple testing. We considered associations with *P*-values below 0.01 (0.05/5) as strong evidence of associations. Results with P-values between 0.01 and 0.05 were regarded as suggestive associations. All analyses were two-sided and conducted using TwoSampleMR (version 0.5.7), Mendelian randomization (version 0.7.0), and MRPRESSO (version 1.0) packages in R software (version 4.2.3). Reporting of the study follows the STROBE-MR statement.

## Results

### UVMR analyses of women’s reproductive traits on the risk of schizophrenia

**Fig. 2:**
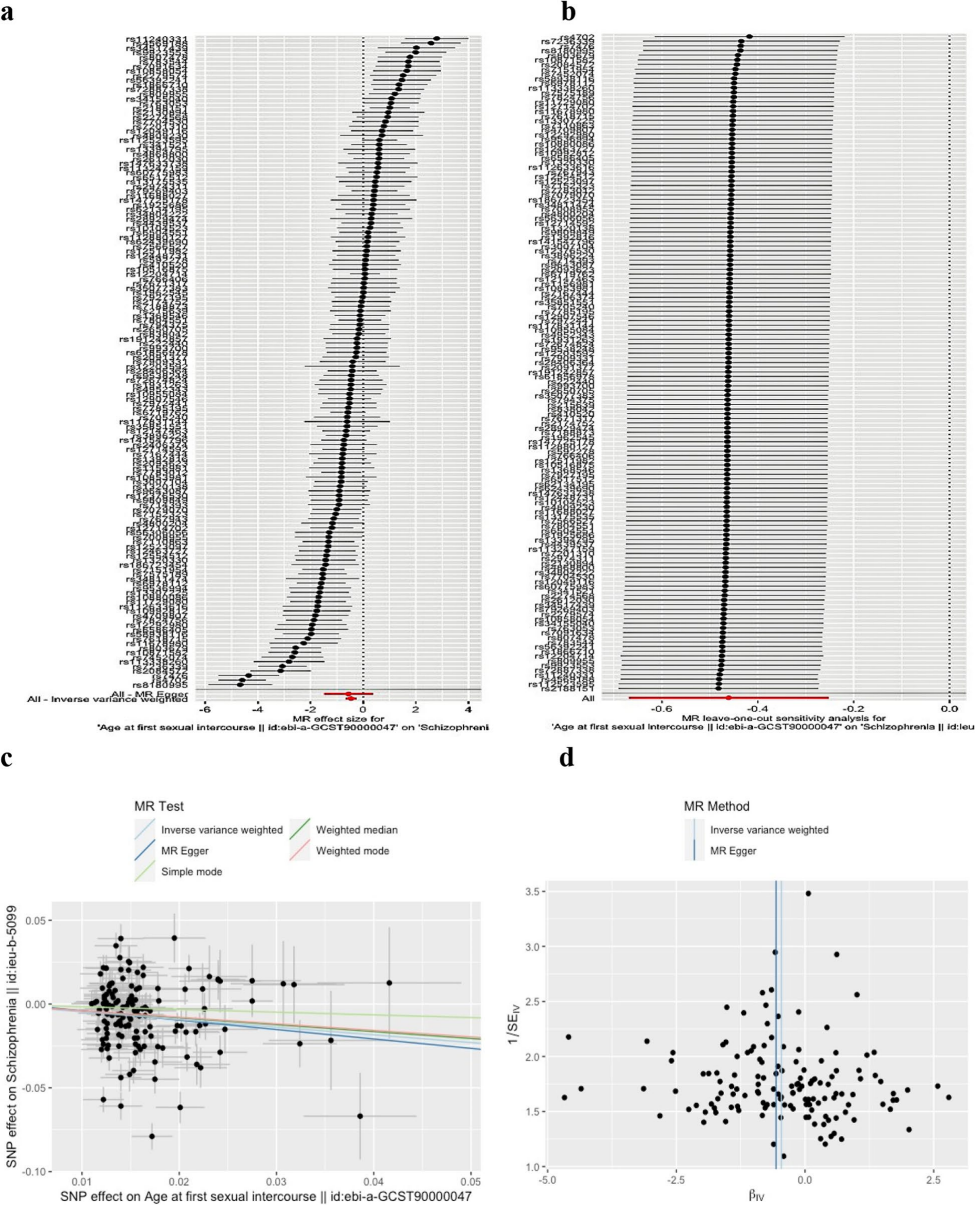
The causal effect of genetically predicted age at first sexual intercourse on the risk of SCZ. **a** Forrest plot. The MR findings of the MR-Egger test and the inverse variance weighted (IVW) method are the significance of the red lines. **b** MR leave-one-out sensitivity analysis. The leave-one-out sensitivity analysis found no single genetic variant-driven causality. **c** Scatter plot. The slope of each line in the SNP scatterplot corresponds to the MR effect estimated by each method, and the slopes differ in magnitude but are in the same direction for the five methods. **d** Funnel plot. The blue line in the SNP funnel plot indicates the IVW estimate and the dark blue line indicates the Mendelian randomization-Egger estimate. No evidence of asymmetry on either side of the blue line of the funnel plot

After LD clumping and deleting pleiotropic SNPs, we chose 62, 176, 66, five, and 107 SNPs as genetic instruments for AFB, AFS, AAM, ALB, and AMP, respectively (Supplementary Tables S1-S5). Women’s reproductive traits displayed mean F statistics ranging from 17.410 to 278.265 (Supplementary Table S6). The primary IVW approach revealed considerable evidence for an inversely causal link between AFS and the risk of SCZ (OR, 0.632; 95% CI, 0.512–0.777; *P* < 0.01; Fig. 2a and c). However, we discovered no evidence that AFB, AAM, ALB, or AMP had a direct influence on the risk of SCZ (all *P* > 0.05; Supplementary Table S7a). The results of the weighted median, MR Egger, simple and weighted models with the IVW method showed no directional pleiotropy, and the MR-Egger intercept test demonstrated no horizontal pleiotropy (Supplementary Table S7b).

### MVMR analyses of women’s reproductive traits on the risk of schizophrenia

In the MVMR, substantial genome-wide genetic variations on BMI, YS, and SI are linked with each woman’s reproductive traits (Supplementary Table S8-S10). After adjusting for BMI, YS, and SI, substantial evidence also shows that genetic susceptibility to AFS has a direct inverse effect on the risk of SCZ (OR, 0.592; 95%CI, 0.407–0.862; *P* < 0.01) (Table 2). Furthermore, the MVMR identified no evidence of causal connections for AAM, AFB, ALB, and AMP (all *P* > 0.05; Table 2). The MVMR-Egger methods produced consistent findings (Table S7a), and the MVMR-median, MVMR-Lasso, and MVMR-Egger intercept tests revealed no horizontal pleiotropy (Supplementary Table S11).

**Table 2:** Association of Reproductive factors with the risk of SCZ using IVW methods in both UVMR and MVMR models

Exposure	Mode	SNPs	*P*-Value	OR	95%LCI	95%UCI
AFB	UVMR	62	0.709	0.982	0.893	1.080
	MVMR	18	0.761	1.017	0.902	1.147
AFS	UVMR	176	1.473*10^−5^	0.632	0.512	0.777
	MVMR	88	6.294*10^−3^	0.592	0.407	0.862
AAM	UVMR	66	0.130	1.085	0.976	1.207
	MVMR	37	0.248	0.926	0.814	1.054
ALB*	UVMR	26	0.224	1.265	0.866	1.848
	MVMR	1	0.156	1.278	0.910	1.794
AMP	UVMR	107	0.344	1.045	0.954	1.143
	MVMR	66	0.619	1.028	0.922	1.146

*AFB* Age at first birth, *AFS* Age at first sexual intercourse, *ALB* Age at last live birth, *AAM* Age at menarche, *AMP* Age at menopause, *MVMR* Multivariable Mendelian randomization, *UVMR* Univariable Mendelian randomization

*P-value cut-offs for instrumental variables are loosened to 1*10^−6^

### Sensitivity analysis

Regarding the sensitivity analysis, MR-Egger, weighted median, simple mode, and weighted mode regression methods were employed to analyze the relationship between IV and outcome (Supplementary Table S7). In addition, forest plots for visualization of relevant results were used (Fig. 2a). Heterogeneity was assessed by the Q test, and heterogeneity (Q, 56.981–721.257; *P* < 0.05) was observed for all IVs. Due to the heterogeneity in the association analysis of AFS-SCZ (Q = 721.257), we applied random effects IVW. In addition, the causal association of AFS-SCZ was found to remain robust after removing each of the SNPs, which was also confirmed by leave-one-out plots (Fig. 2b). Potential polymorphic outlier SNPs were detected in the IVs-SCZ association using MR-PRESSO. Although outlier SNPs were present in the AFS-SCZ association, the causal association remained robust after the removal of the outlier SNPs (*P <* 0.01). Pleiotropy was not detected in the AFS-SCZ association pleiotropic test (*P* = 0.824). Funnel plots of the AFS-SCZ association were generally symmetrical, which was another indication of no horizontal pleiotropy (Fig. 2d).

## Discussion

In the current study, we investigated the causal relationship between five female reproductive traits (AAM, ALB, AFB, AFS, and AMP) and the risk of SCZ. We utilized summary statistics from the largest GWAS conducted on these reproductive traits in a population of European ancestry and constructed robust instruments using exposure-related SNPs. Furthermore, we found a causal association between AFS-SCZ using UVMR analysis. The sensitivity analysis of the aforementioned results to other MR methods was robust and showed no evidence of horizontal pleiotropy. Notably, although the effect of AFS on the risk of SCZ was attenuated in the MVMR analysis, it was still statistically significant. Therefore, we conclude that premature female sexual activity increases the risk of SCZ in the largest sample size of the European population.

Adolescence and teenage years are known to prove to be a sensitive period for psychological and behavioral problems. This period of intense hormonal fluctuations is associated with a high incidence and worsening of potential mental illness [44]. From a gender perspective, women are more likely to have psychological complaints than men during that period [45]. Accordingly, first menstruation is going to be used as a valid factor for investigation of female puberty problems. In some studies, the early age of menarche has been found to be associated with mental health and behavioral problems [46]. Psychotic symptoms and suicidal and non-suicidal self-harming behaviors were more prevalent in students with early menarche than in those with on-time and delayed menarche [47]. However, the results of the current study did not find a causal association between age at menarche and the risk of SCZ. The findings of this study are consistent with some previous research [48, 49]. Therefore, the relationship between the two aforementioned factors remains controversial and needs to be further explored by further study. An equally controversial topic over the last several decades has been the relationship between menopause and SCZ [50, 51]. In contrast to men, women have a second peak in the onset of SCZ after the age of 40*–*45 years, which is associated with the onset of perimenopause in their lives [52]. As compelling evidence in support of the above, late-onset SCZ has been documented to be more frequent and more severe in women than in men [53, 54]. To explain the above phenomenon, the theory of menopausal risk has been proposed, which has been focused on the potential role of ovarian hormones (i.e., estrogen, progesterone) in women’s risk of psychiatric disorders in midlife [55]. In addition, there is growing evidence that (1) estrogen has a protective effect on neurons [56], (2) estrogen has been hypothesized to have an antidopaminergic effect [50], and (3) periods of the abrupt decrease in estrogen levels during a woman’s menstrual cycle and life cycle are associated with increased psychotic symptoms and vice versa [57]. Nevertheless, our research failed to find a causal link between AMP-SCZ by means of IVs. For the current scenario, we consider that menopausal status cannot be reliably predicted by age [58, 59]. Hence, age at menopause, as a proxy for its change, may be inadequate.

As society progresses and human civilization advances, we need to face a worldwide delay in reproduction [60]. There has also been extensive discussion about the relationship between parental age at childbirth and mental disorders [61–63]. It is well known that increasing maternal age at childbirth raises the risk of chromosomal segregation errors [64]. Moreover, there is growing recognition that maternal age at childbirth is associated with mental disorders [10, 11]. A relatively recent study by McGrath et al. (2014) [65] conducted a comprehensive analysis using household data extracted from the Danish Central Registry of Psychiatry and reported a U-shaped relationship between maternal age and the risk of SCZ. It was found that children of older mothers had a higher risk of the illness compared to younger mothers (25*–*29 years). In their second analysis, the risk of SCZ tended to be highly correlated with the age of the mother, controlling for the age of the father. Subsequently, the above findings were re-validated from the perspective of genetic association [17]. However, we failed to identify a causal association in females with SCZ regardless of the age of the first or last birth. A possible explanation may be the relatively small number of IVs selected for this study, and the existence of complex social, psychological, and biological effects between the age of the first or last birth and the risk of SCZ.

Information about people’s sexual and reproductive behaviors is rarely mentioned publicly, despite the fact that these behaviors play a prominent role in determining social and emotional well-being [66]. Sexual intercourse usually begins at puberty, with the first experiences for males and females beginning at approximately 16.8 and 17.2 years of age (https://kinseyinstitute.org/research/faq.php.). Unfortunately, accumulating evidence suggests that the onset of adolescent sexual activity has become earlier [67, 68]. Early sexual initiation is defined as the first sexual intercourse occurring before the age of 15 [69]. Early sexual intercourse is not only associated with having multiple sexual partners, inconsistent condom use, sexually transmitted infections, and unwanted pregnancies [70, 71], but also with mental health [72, 73]. A recent research study [74] selected data from the Seattle Social Development Project in Washington State (*n* = 808) and used linear logistic regression to analyze the strong association between early sexual initiation and poor mental health. The findings were consistent with a review that selected 28 studies [75]. Nevertheless, large cross-sectional studies involving 60,040 adolescents have expressed concern about the relationship between the two [76]. More importantly, one bidirectional two-sample Mendelian randomization study reconfirmed that, genetically, premature sexual behavior is a risk for major depression [77]. Similarly, the current study found a causal association between premature sexual intercourse and the risk of SCZ. In addition, given the strong associations between smoking [41], educational achievement [78] with SCZ, and female reproductive characteristics, the MVMR approach revalidated these findings, controlling for relevant factors. That is, early female sexual intercourse increases the risk of SCZ.

The potential mechanisms driving the causal link between premature sexual behavior and the susceptibility to SCZ may not follow a straightforward path. It is well known that adolescence is a sensitive time for physical development and hormonal fluctuations. As a mental health problem, behavioral difficulties occur in one in four adolescents [79] and can be categorized into externalizing and internalizing behavior problems. Adolescents with externalizing behavior problems are more likely to exhibit aggressive and risk-taking behaviors, while those with internalizing behavior problems exhibit withdrawal and depression [80]. Previous studies have reported that compared to others, adolescents with internalizing and externalizing symptoms are more likely to engage in early sexual activity [81, 82]. Early sexual activity leads to internal guilt, low self-esteem, bullying, and social stigma, which contribute to symptoms such as anxiety, depression and suicidal thoughts, and psychological distress [83]. In terms of psychosocial influences, parental and family factors have a significant impact on the age of sexual initiation of adolescents. Family factors associated with early sexual behavior include single-parent family structure, poor parent-adolescent relationships, low levels of parental supervision, and permissive parental attitudes toward sexual activity in previous studies [84, 85]. Another study analyzing school-age children in 50 countries found that parental supervision had a significant impact on reducing the odds of early sexual behavior among adolescents, especially for girls [86]. Based on bioecological theory, an analysis from the perspective of resilience shows that protective processes include adolescent academic performance, expectations, and school approval, in addition to close mother-child and father-child relationships, parental supervision, and family routines. Moreover, risk factors include criminal behavior and dangerous neighborhood environments, as well as the many factors mentioned above [87]. Perhaps active sexual health education for adolescents also needs to be accompanied by a multifaceted approach involving schools, families, and society, so as to promote adolescent mental health and reduce the risk of SCZ.

Early study has reported that the heritability of SCZ is as high as 80% [88]. Discussion has also proposed that SCZ is essentially a genetic disorder [89]. Therefore, it is necessary to explore the influence of female reproductive characteristics on SCZ from the perspective of genetic susceptibility. Although discussion [90] on this issue has been conducted, it has been mainly limited to gestational health. However, our study opted for five indicators that possess broader applicability and better represent women. Furthermore, this study used GWAS data related to a larger number of patients with SCZ, and modifiable risk factors were incorporated into the analysis in order to identify additional mechanisms of intervening in the development of the disease. Nevertheless, our research inevitably has some limitations. First, in our study, the GWAS data only corresponded to individuals of European ancestry. Therefore, the applicability of the study findings may be limited. Second, when we screened the IVs, numerous indicators of female reproductive characteristics, such as pregnancy loss, number of miscarriages, and number of stillbirths that did not meet the requirements, were not included. The subsequent generation of a new, large GWAS database may provide a more comprehensive assessment of the relationship between the two. Third, although we screened and controlled for the influence of some factors regarding the relationship between female reproductive characteristics and the risk of SCZ, reproductive behavior is complex, since it is influenced by different components including genetic, environmental, and socioeconomic factors, as well as their complex interactions. A limited number of factors, however, have been discussed in this study. Fourth, our study addressed the issue of female reproductive characteristics and genetic susceptibility to SCZ, while GWAS data for outcome variables and confounders failed to separate the male dataset. This increases the possibility of false-negative findings and caution should be exercised when generalizing the results of the study. Finally, racial differences may influence genetic susceptibility to SCZ. Given that our outcome variable involves multiple ethnic ancestries, our study has not yet ruled out the influence of this factor on the findings.

In summary, our findings provide convincing evidence that early AFS is a risk factor for SCZ. Promoting and educating adolescents about good sexual health may be an effective way to reduce the risk of SCZ.

### Supplementary Information

**Additional file 1:** Supplementary materials associated with this article can be found online at **Table S1-S11**.

## Data Availability

Data sources and methods for working with these data are detailed in the Materials and Methods and the Supplementary Tables; all data utilized in this investigation are freely available to the public. To facilitate communication, we have shared the R code for this study on GitHub (https://github.com/Forworks0410/a-flash-of-insight). Further details can be provided upon reasonable request by contacting the corresponding author.

## Abbreviation

AAM: Age at menarche
AD: Alcoholic drinks per week
AFB: Age at first birth
AFS: Age at first sexual encounter
ALB: Age at last live birth
AMP: Age at menopause
BMI: Body mass index
CI: Coffee intake
GWAS: Genome-wide association studies
IVs: Instrumental variables
IVW: Inverse-variance weighted
LD: Linkage disequilibrium
MR: Mendelian randomization
MVMR: Multivariable Mendelian randomization
PA: Physical activity
PGC: Psychiatric Genomics Consortium
SCZ: Schizophrenia
SNPs: Single nucleotide polymorphisms
SI: Smoking initiation
SSGAC: Science Genetic Association Consortium
UVMR: Univariable Mendelian randomization
YS: Years of schooling
